# Using structural equation modeling to assess pathways between structural stigma and tobacco use among sexual and gender minority young adults living in the United States

**DOI:** 10.21203/rs.3.rs-5417843/v1

**Published:** 2024-12-17

**Authors:** Wilson Figueroa, Srini Sridhar, Emma Jankowski, Alysha Ennis, Anne Trinh, Eric Seiber, Joanne Patterson

**Affiliations:** The Ohio State University; The Ohio State University; The Ohio State University; The Ohio State University; The Ohio State University; The Ohio State University; The Ohio State University

**Keywords:** Structural Stigma, Tobacco use, Economic Stability, Sexual and Gender Minorities, Structural Equation Modeling

## Abstract

**Background:**

Sexual and gender minority young adult (SGM YA) populations use tobacco at higher rates than their non-SGM YA peers. Prior studies have identified significant correlations between interpersonal stigma and tobacco use, yet structural stigma may also influence tobacco use among SGM YA. This study aimed to assess the indirect effects of structural stigma on current tobacco use among SGM YA and non-SGM YA via depletion of economic resources, interpersonal discrimination, and perceived psychological stress.

**Methods:**

Structural Equation Modeling was used to conduct a secondary data analysis from a cross-sectional parent study. Eligible participants were 18–35 years old and currently residing in the U.S. (N = 2,857). Current use of combustible cigarettes and nicotine vapes was our dependent variable. Our independent variable of interest, structural stigma, was a latent variable comprised of three state-level indicator items: Attitudes toward SGM people, SGM protective policies (absence of), and SGM discriminatory policies (introduced or passed in 2022). We assessed three mediators of interest: Depletion of economic resources was a latent variable, which included two indicator items: food insecurity and financial strain. Interpersonal discrimination and perceptions of psychological stress were also assessed. Covariates included race/ethnicity, age, and educational attainment.

**Results:**

Structural stigma was indirectly associated with current tobacco use via depletion of economic resources for SGM YA, but not non-SGM YA. Interpersonal discrimination was also directly and indirectly associated with current tobacco use via depletion of economic resources for both groups.

**Conclusions:**

Future tobacco intervention research should consider the role of structural stigma when working with SGM YA; specifically, how interventions promoting economic stability may influence tobacco use and cessation in this population.

## BACKGROUND

Sexual and gender minority young adult (SGM YA) populations use tobacco at significantly higher rates than their non-SGM YA peers, with disparities beginning in adolescence and continuing into young adulthood.[1–3] Minority stress theory purports that the unique multilevel stressors experienced by SGM YA lead them to engage in risky health behaviors (such as nicotine vape and cigarette use) at higher rates.[4] Prior studies have identified significant correlations between minority stressors and tobacco use; however, most of these studies have focused on individual and interpersonal stigma, including experiences of internalized queerphobia and perceived discrimination.[5] Yet, structural or societal-level stressors may also influence tobacco use among SGM YA.

Structural stigma has been defined as “societal-level conditions, cultural norms, and institutional policies that constrain the opportunities, resources, and wellbeing” of minoritized populations.[6] In 2023, there were 510 anti-SGM bills introduced in state legislatures in the United States (US), a three-fold increase from the previous year. This trend is continuing in 2024, with 527 anti-SGM bills introduced into state legislatures to date. These bills span a variety of issues, from laws prohibiting free speech and expression to those limiting healthcare access for SGM people.[7] A recent narrative review of quantitative studies examining the effect of structural stigma on SGM health found that, to date, 14 studies have examined how structural stigma is associated with tobacco use among SGM.[8] These studies examined a diverse set of exposures and reported mixed results. Seven studies found that protective structural factors (e.g., community supportiveness, anti-discrimination laws) decreased rates of smoking among SGM youth and young adults.[9–15] An additional three studies found that greater levels of structural stigma (e.g., negative community attitudes, discriminatory policies) were associated with higher rates of tobacco use.[16–18] The remaining studies demonstrated mixed effects. For example, a study of US adults indicated that legalization of same sex relationships led to higher smoking among women in same sex households but not men.[19] None of these studies examined mediating mechanisms by which structural stigma may influence tobacco use among SGM YA.

Ecological models of population health suggest that upstream factors, such as living in SGM discriminatory contexts (i.e., experiencing structural stigma), deplete the economic resources available to SGM people and increase interpersonal discrimination experiences, ultimately leading to increased psychological stress and maladaptive coping behaviors.[20] Previous work supports this hypothesis, though this work is sparse, with only 10% of studies examining structural stigma and its relation to health having examined mediating effects.[8] The extant literature has predominantly explored interpersonal and individual-level minority stress (e.g., discrimination experiences, internalized queerphobia) as candidate mechanisms between structural stigma and mental health outcomes.[8] Generally, these studies find that SGM youth and adults living in states with high structural stigma (vs low structural stigma) experience greater interpersonal and internalized minority stress, which is associated with poor mental health. No studies examined economic factors as candidate mechanisms between structural stigma and poor health outcomes. However, in one study of gay men, individuals living in states with greater structural stigma (defined as prejudicial attitudes) reported decreased wages, and prejudice coming from their workplace managers was found to mediate this association.[21] Together, this body of theoretical and empirical research suggests that structural stigma may be influencing health outcomes among SGM people through several mediating mechanisms.

### Purpose of the Study

This study builds on the limited evidence testing the mechanisms through which structural stigma influences SGM health behaviors. We aimed to assess the indirect effects of structural stigma on current tobacco use among SGM YA and cisgender heterosexual YA (non-SGM YA). Consistent with ecological and minority stress models, we examined the potential pathways that may create and exacerbate disparities in tobacco use. Specifically, we were interested in how the depletion of economic resources, interpersonal discrimination, and perceived psychological stress might mediate the relationship between structural stigma and current tobacco use among SGM and non-SGM YA.

## METHODS

### Study Design

This study is a secondary data analysis from a larger, cross-sectional parent study meant to evaluate the effectiveness of tobacco public health education messages among young adults. Prolific ( https://www.prolific.co/), an online subject recruitment platform, was used to recruit participants, due to its large access to US nationals (~ 38.000), its base of young adults (approximately one-third are < 35 years old), and its effectiveness for reaching minoritized populations, including SGM people. The parent study purposively oversampled SGM people, people who smoke cigarettes, and people who vape nicotine. We also sampled proportionally for each racial/ethnic group per US Census 2020 estimates. Potential participants were prescreened via Prolific. Those who met the eligibility criteria of being aged 18–35 years old and currently residing in the US were directed to an online consent form. After consenting, participants (N = 2,857) were directed to a Qualtrics survey. They received $4.50 for participating. The parent study was approved by The Ohio State University Institutional Review Board (2021C0020).).

### Measures

Measures are described in detail in Table 1. Our sample included two subgroups of interest: SGM YA and non-SGM YA. We used two items to assess participants’ self-reported sexual identity group and self-reported gender identity group. Individuals identifying with sexual or gender minority identity groups (e.g., lesbian, gay, bisexual, queer, transgender, nonbinary) were defined as SGM; those identifying with cisgender and heterosexual groups were defined as non-SGM. Our dependent variable, current tobacco use, was a latent variable comprised of two indicator items: past 30-day use of combustible cigarettes or past 30-day use of nicotine vapes. Our independent variable of interest, structural stigma, was a latent variable comprised of three state-level indicator items: Attitudes toward SGM people, SGM protective policies (absence of), and SGM discriminatory policies (introduced or passed in 2022). We assessed three mediators of interest: Depletion of economic resources was a latent variable, which included two indicator items. The first assessed past 12-month experiences of food insecurity and the second assessed perceived financial strain. Interpersonal discrimination was assessed using the everyday discrimination scale, which assesses self-reported frequency of daily discrimination experiences in social situations.[22] We used a general measure of discrimination rather than assessing SGM-specific discrimination experiences so that we could assess the influence of this hypothesized mediator on tobacco use among non-SGM YA. Finally, the four-item Perceived Stress Scale was used to assess individual perceptions of psychological stress.[23] Covariates included race/ethnicity, age, and educational attainment.

### Analysis

A comprehensive investigation was conducted using Structural Equation Modeling (SEM) to examine the association between structural stigma and current tobacco use, including combustible cigarettes and nicotine vapes. This study employed SEM with Maximum Likelihood Estimation (MLE) to examine the relationship between multiple variables, considering their direct and indirect effects on current tobacco use. All SEM analyses were performed in R (V 4·2·2) using the Lavaan package (0·6–16). Modification indices were inspected for significant areas of model misfit; however, none were detected so model adjustment was not necessary. Model fit was evaluated using the following fit indices: Comparative Fit Index (CFI), Root Mean Square Error of Approximation (RMSEA), and Standardized Root Mean Squared Residual (SRMR). Indicators of acceptable model fit were considered as CFI > 0·9, RMSEA < 0·06, and SRMR < 008. An alpha of ·05 was used to indicate statistically significant pathways between constructs.

## RESULTS

### Sample Description

As shown in Table 2, across both groups (n = 1,288 SGM and n = 1,368 heterosexual), most participants were White (64% SGM, 55% non-SGM) and had similar current use of nicotine vapes (35% SGM, 36% heterosexual). More non-SGM than SGM YA were Black, Indigenous, and People of Color (BIPOC; 45% vs 36%), aged 25–35 years (31% vs 26%), held a four-year degree or higher (53% vs 47%), and currently smoked combustible cigarettes (31% vs 26%). SGM YA reported greater financial strain (M = 5·35 vs. M = 4·62), food insecurity (M = 4·24 vs. M = 3·62), interpersonal discrimination (M = 11·98 vs. 10·47), and perceived stress (M = 8·61 vs. 7·26) compared to non-SGM YA.

### Preliminary analyses

Bivariate correlations among all measured variables are presented in Table 3. Among non-SGM YA, our indicator variables of structural stigma (attitudes towards SGM individuals, SGM discriminatory bills, absence of SGM protective policies) were not significantly associated with our outcome variables (current combustible cigarette and nicotine vape use). Other than financial strain (r = 0·06, p < 0·05), our indicator variables of structural stigma were not related to mediators nor covariates. However, there was a significant positive intercorrelation between our measures of financial strain, food insecurity, interpersonal discrimination, and perceived stress. Finally, all covariates (age, race, and education) were significantly associated with our outcome variables. For race, being a race other than White was associated with increased current use of combustible cigarettes and nicotine vapes. For education, having less than a four-year degree was associated with increased current use of combustible cigarettes and nicotine vapes. For age, being between 25–35 years was associated with greater current use of combustible cigarettes, while being between 18–24 years was associated with greater current use of nicotine vapes. Latinx ethnicity was not significantly associated with our outcome variables among non-SGM YA.

Among SGM YA, like non-SGM YA, none of our indicator variables of structural stigma were directly associated with current use of combustible cigarettes or nicotine vapes. However, discriminatory bills under consideration were positively associated with financial strain and enacted discriminatory bills were positively associated with food insecurity. There was a significant positive intercorrelation between our measures of financial strain, food insecurity, experiences of interpersonal discrimination, and perceived stress. Finally, like non-SGM YA, being between 25–35 years was associated with greater current use of combustible cigarettes, while being between 18–24 years was associated with greater current use of nicotine vapes for SGM YA. No other covariates were associated with outcome variables among the SGM group.

### Minority Stress Model:

**Figure 1.**
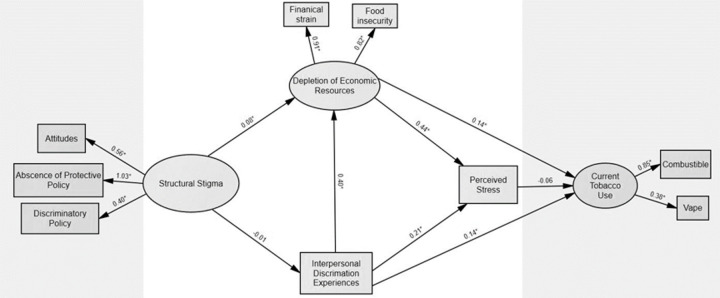
Structural Equation Model assessing theoretically informed pathways between structural stigma and currenttobacco use among sexual and gender minority (SGM) young adults

**Figure 2.**
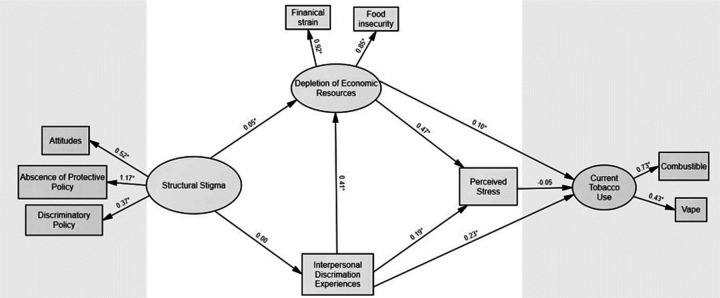
Structural Equation Model assessing theoretically informed pathways between structural stigma and currenttobacco use among cisgender heterosexual (non-SGM) young adults

SEM was used to test the hypothesized model (Figs. 1 and 2), examining the indirect effects of structural stigma (i.e., discriminatory attitudes, discriminatory policies, and absence of protective policies) on current tobacco use among subpopulations of SGM and non-SGM YA. Depletion of economic resources (i.e., food insecurity and financial strain) and perceived discrimination experiences were assessed as mediators between structural stigma and current tobacco use. The data fit for both SGM YA (χ^2^ = 267·5, p < 0·05, CFI = 0·92, SRMR = 0·05, RMSEA = 0·06; Fig. 1) and non-SGM YA (χ^2^ = 234·6, p < 0·05, CFI = 0·95, SRMR = 0·05, RMSEA = 0·05; Fig. 2).

### Pathways between structural stigma and current tobacco useamong SGM YA

The final model accounted for 5.5% of the variance in current tobacco use among SGM YA. Structural stigma was indirectly associated with greater current tobacco use through depletion of economic resources for the SGM YA group (β = 0·01, z = 2·16, p < 0·05, Table 4). This indicates that greater structural stigma is associated with greater depletion of economic resources which, in turn, is associated with increased current tobacco use among our sample of SGM YA.

### Pathways between interpersonal discrimination and currenttobacco use among SGM YA

Experiencing interpersonal discrimination was both directly and indirectly, via depletion of economic resources, associated with greater current tobacco use among SGM YA. As seen in Table 4, the indirect effect of interpersonal discrimination on current tobacco use was statistically significant (β = 0·06, z = 3.01, p < 0·01).

### Pathways between perceived stress and current tobacco useamong SGM YA

Depletion of economic resources and experiencing interpersonal discrimination were also both directly associated with perceived stress, yet there was no association between perceived stress and current tobacco use for SGM YA.

### Pathways between structural stigma and current tobacco useamong non-SGM YA

The final model accounted for 11·7% of the variance in current tobacco use for the non-SGM YA group. While structural stigma was significantly associated with depletion of economic resources among non-SGM YA, there was not a significant indirect pathway linking structural stigma and current tobacco use via depletion of economic resources for non-SGM YA.

### Pathways between interpersonal discrimination and currenttobacco use among non-SGM YA

Experiencing interpersonal discrimination was both directly and indirectly, via depletion of economic resources, associated with greater current tobacco use among non-SGM YA. As seen in Table 4, the indirect effect of interpersonal discrimination on current tobacco use was statistically significant (β = 0·04, z = 2·12, p < 0·05).

### Pathways between perceived stress and current tobacco useamong non-SGM YA

Depletion of economic resources and experiencing interpersonal discrimination were also both directly associated with perceived stress, yet there was no association between perceived stress and current tobacco use for non-SGM YA.

### Covariate associations with current tobacco use

In terms of covariates, age was significantly associated with current tobacco use for non-SGM YA, indicating that individuals ages 25–35 years were more likely to use tobacco compared to those aged 18–24 years. Additionally, race was associated with current tobacco use only for non-SGM YA, indicating that more BIPOC YA in this group reported current tobacco use more than White YA.

## Discussion

Our study is one of the first to assess how structural stigma may influence current tobacco use among SGM and non-SGM YA via theoretically informed pathways. Results indicated that structural stigma was indirectly associated with current tobacco use through the depletion of economic resources (increased financial strain and food insecurity) for SGM YA, but not for non-SGM YA. Additionally, interpersonal discrimination was both directly and indirectly, via depletion of economic resources, associated with current tobacco use for both SGM and non-SGM YA. Previous research examining food insecurity among SGM individuals has found that states with Religious Freedom Restoration Acts or “religious freedom” laws allow institutions, including food pantries, to deny services to select community members, such as SGM YA, based on religious beliefs.[24] Such laws may contribute to the higher rates of food insecurity seen in SGM compared to non-SGM individuals.[25] Food insecurity has been linked to tobacco use in a variety of vulnerable populations.[26] Although previous research has indicated that the direction of the association is unclear (i.e., causality cannot be established given that the majority of studies, including the present one, have been cross-sectional in nature), both financial strain and stress have been suggested as possible mechanisms that link food insecurity to tobacco use.[26] Longitudinal studies are needed to further examine the directionality of the association between food insecurity and tobacco use. Nevertheless, our results indicate depletion of economic resources may be one mechanism by which structural stigma and interpersonal discrimination influence current tobacco use among SGM YA. Given the well documented disparities in tobacco use among SGM YA, future tobacco intervention studies should consider food insecurity and financial strain as points of intervention for this population.

In a recent narrative review examining the role of structural stigma on health outcomes (including behavioral health), a conceptual model was proposed indicating that interpersonal discrimination may mediate the association between structural stigma and behavioral health (i.e., current tobacco use) outcomes.[8] However, our study is one of the first to examine the pathway proposed by this conceptual model (i.e., structural stigma →interpersonal discrimination→behavioral health outcomes). In our study, structural stigma was not indirectly associated with current tobacco use via interpersonal discrimination. This null finding for an indirect pathway between structural stigma and current tobacco use via interpersonal discrimination may be due to construct operationalization. While our structural stigma measure was SGM-specific, our interpersonal discrimination measure assessed perceptions of interpersonal discrimination for any reason (e.g., due to SGM identity, race/ethnicity, religion body size, physical ability, etc.) so that we could assess this construct among non-SGM YA. However, similar to prior studies[5], we identified a direct association between interpersonal discrimination and current tobacco use. As mentioned previously, longitudinal studies are needed to examine how structural stigma may influence interpersonal discrimination over time and how this, in turn, may influence current tobacco use among SGM YA. This work should include measures assessing SGM-specific interpersonal discrimination, which may be more closely associated with structural stigma.

Our study has limitations, including the use of cross-sectional data and the lack of a sufficient sample size to examine the SGM YA subgroup (e.g., bisexual vs gay, or by gender identity) as a moderator. Nevertheless, our study addressed several gaps identified previously in the literature and is one of the few to conduct mediational analyses examining the indirect effects of structural stigma on health outcomes among SGM YA. Previous research[17,18] indicates a need to examine how structural stigma may indirectly influence tobacco use among SGM individuals, specifically, and our study is one of the first to examine this association as it relates to current tobacco use in SGM YA. Our study also addresses a gap identified in the literature[8] by including a non-SGM YA comparison group. In doing so, we demonstrated that structural stigma is associated with current tobacco use, via depletion of economic resources, for SGM YA only. Given unprecedented increases in legislation targeting SGM rights, identifying intervention points to attenuate the negative effects of structural stigma on tobacco use is critical for promoting SGM health across the life course.

## Conclusions

In conclusion, although the association between structural stigma and health in general has been well established among SGM, our study is one the first to examine the indirect effects of structural stigma on current tobacco use specifically among a highly vulnerable population, SGM YA. Future tobacco cessation and harm reduction intervention research should consider the role of structural stigma when working with SGM YA; specifically, how interventions promoting economic stability may influence tobacco use and cessation in this population. It will also be important to examine how changes in general attitudes towards SGM individuals and lack of protective policy and discriminatory policy toward SGM individuals influences tobacco use in SGM YA over time, as previous research has indicated that improvements in structural stigma may be associated with improvement in life satisfaction in this population.[27]

## Figures and Tables

**Table 1.** Survey Measures

Variable	Question	Coding
**Independent Variable:** Structural stigma		
Attitudes toward sexual minoritized people^25^	1. “Homosexual couples should have the right to marry one another” 2. “If somebody in your community suggested that a book in favor of homosexuality should be taken out of your public library, would you favor removing this book, or not?” 3. “Should a man who admits that he is a homosexual be allowed to teach in a college or university, or not?” 4. “Suppose a gay person wanted to make a speech in your community. Should he be allowed to speak, or not?” 5. “What about sexual relations between two adults of the same sex –do you think it is always wrong, almost always wrong, wrong only sometimes, or not wrong at all?”	1 = Level of anti-gay prejudice at the regional-level (indicated by percent negative responses to items) was above the average anti-gay prejudice at the US national-level. 0 = Indicated that the level of anti-gay prejudice at the state-level was below the US national level. Three of the four questions were already written as dichotomous, such that we could compare the percent of negative responses to those questions at the regional level to the national level. Two questions required conversion to a dichotomous response to ensure consistency across the five items. For the first item, related to homosexual sexual relations, we summed the percent of responses indicating that same-sex relations were wrong at least to some degree before comparing the percent of prejudicial responses at the regional- and national-levels. For the second item, related to the rights of homosexual couples to marry, we summed the percent of responses indicating that homosexual relations were wrong at least to some degree before comparing the percent of prejudicial responses at the regional- and national-levels. We then summed the five anti-gay prejudice questions into a single summed value (0–5) that represented the sum prejudicial attitudes at a regional level. These data are only available at the aggregate level by region, so the results from each region were extrapolated to individual states to give a state-level estimate of anti-gay prejudice.
Absence of Protective Policies^26^	Protective policies for SGM people include protections against employment discrimination, housing discrimination, discrimination in public accommodations, credit discrimination, and discrimination against state employees. Different states have differing policies protecting SGM people from these kinds of discrimination. The Movement Advancement Project collected and summarized these policies by state. States included some policies that only protected lesbian, gay, and bisexual (LGB) individuals, while some also included protections for transgender individuals.	1 = The absence of a law in 2022 that protected against discrimination based on gender identity in each of the 5 domains as a “1” (range 0–5). We also coded the absence of a law that protected projected against discrimination based on sexual orientation in each of the five domains as a “1” (range 0–5). 0 = Presence of a protective law These two subscales were summed to create a total score (0–10) representing the lack of absence of SGM protective policies at the state-level.
Recently Introduced or Passed Discriminatory State Policy^27^	Differing bills targeting SGM rights have been under consideration and enacted in state-level legislatures across the country. We gathered data from the American Civil Liberties Union (ACLU) documenting the PRESENCE of discriminatory bills that were introduced and which died, were referred to committee or the governor, or were introduced and signed into law in the 2022 legislative session. These bills centered around ten topical areas across two domains: Anti-Transgender Bills (these bills included: restricting healthcare for transgender youth, single-sex facility restrictions, excluding transgender youth from athletics, other school or curriculum restrictions, restrictions on accessing accurate ID, and other bills that target transgender and nonbinary people for discrimination) and Religious exemption Bills (these bills included: Religious Freedom Restoration Acts, Religious exemptions in healthcare implicating SGM people, religious exemptions in adoption and foster care, and other religious exemption bills).	1 = The introduction of a bill in 2022 in each topical area across the three domains in the state (range 0–10) And/or 1 = Adoption AND signing into law of a bill in 2022 in each of the topical areas in the state (range 0–10). 0 = No law passed or introduced in that topical area in the state These two subscales were summed to create a total score (0–20) representing the presence of SGM discriminatory policies introduced in the 2022 legislative session.
**Mediators**		
Depletion of economic resources		
Food Security^28^	The questions in this scale ask you about your feelings and thoughts during the past 12 MONTHS. In each case, please indicate how often you felt or thought a certain way. 1. How often in the past 12 months, were you worried or stressed about having enough money to buy nutritious meals? 2. How often in the past 12 months did you worry your food would run out before you got money to buy more? 3. How often in the past 12 months, did the food you bought not last and you didn’t have money to get more?	Answer options ranged from 1 (Never) to 5 (Very Often). Responses were summed (Cronbach’s alpha: SGM = 0.90, non-SGM = 0.91 Range: 3–15)
Perceived Financial Stress	The questions in this scale ask you about your feelings and thoughts during the past 12 MONTHS. In each case, please indicate how often you felt or thought a certain way. 1. How often in the past 12 months, were you worried or stressed about having enough money to pay your rent or mortgage? 2. How often in the past 12 months, were you worried or stressed about having enough money to pay for needed medical care or medication? 3. How often in the past 12 months, were you worried or stressed about having enough money to pay other bills (for example: phone, car, credit cards)?	Answer options ranged from 1 (Never) to 5 (Very Often). Items were summed to construct a single measure of financial strain (Cronbach’s alpha: SGM = 0.86, non-SGM = 0.88; Range: 3–15)
Interpersonal Discrimination ^29^	In your day-to-day life, how often do any of the following things happen to you? 1. You are treated with less courtesy or respect than other people are. 2. You receive poorer service than other people at restaurants or stores. 3. People act as if they think you are not smart. 4. People act as if they are afraid of you. 5. You are called names or insulted. 6. You are threatened or harassed.	Answer options ranged from 1 (Never) to 5 (Almost every day). Items were summed and averaged (Cronbach’s alpha = SGM = 0.84, non-SGM = 0.86; Range 1–5).
Perceived Stress^30^	The questions in this scale ask you about your feelings and thoughts during the last month. In each case, please indicate how often you felt or thought a certain way. 1. In the last month, how often have you felt that you were unable to control the important things in your life? 2. In the last month, how often have you felt confident about your ability to handle your personal problems? 3. In the last month, how often have you felt that things were going your way? 4. In the last month, how often have you felt difficulties were piling up so high that you could not overcome them?	Answer options ranged from 1 (Never) to 5 (Very Often). Items were summed and averaged (Cronbach’s alpha = SGM = 0.83, non-SGM = 0.81; Range 1–5).
Demographics		
Race/ethnicity	What racial and/or ethnic groups do you identify with? (Check all that apply) ○ Asian ○ Black/African American ○ Hispanic/Latinx/Latino/Latina ○ Middle Eastern ○ Native American or Alaskan Native ○ Pacific Islander ○ White/Caucasian ○ I use other words to describe my race and ethnicity	0 = “White/Caucasian” 1 = “BIPOC+” (all other response options, including those that selected multiple races)
Age	How old are you? (years)	This was an open response option. Responses were transformed into a binary categorical variable. 0=18–24 years 1=25–35 years
Education	demo_educ What is your highest grade completed? ○ 11th grade ○ High school diploma or GED ○ Technical school (Vocational Technical, Career Certificate, etc.) ○ Some college (not graduated) ○ Associate’s Degree ○ Bachelor’s Degree ○ Master’s Degree ○ Doctoral Degree or other terminal Professional Degree (e.g. MD, JD)	0 = No four-year degree 1 = Four-year degree or higher
Sexual Orientation and Gender Identity	We know that sexuality and gender are complex and fluid. One challenge with research is that we need to group people into a smaller number of categories for statistical analyses. **We think it is important to include LGBTQ people of diverse sexual and gender identities, but we don’t want to assign you to a group that doesn’t feel representative of who you are.** So, we want you to tell us: **If you had to be counted in one sexual identity and one gender identity group, what would you choose?** If I had to choose, my sexual identity group would be: ○ A lesbian / gay category ○ A bisexual / bi+ / pansexual / plurisexual category ○ A heterosexual category ○ An asexual category ○ Unsure because...(please specify) If I had to choose, my gender identity group would be: ○ A **trans/transgender** category (usually refers to people who were assigned a sex and gender at birth that does not accurately represent them) ○ A **cisgender** category (usually refers to people who endorse the same sex and/or gender they were assigned at birth) ○ A **nonbinary** category ( ususally refers to someone who has an identity other than exclusively woman/female or man/male)	0 = Cisgender and Heterosexual 1 = SGM (participants who described their sexual identity group as lesbian, bisexual, or asexual, or participants who described their gender identity group as transgender or nonbinary)
**Dependent Variable**: Current Tobacco use		
**Nicotine vaping**	Have you ever used a nicotine vape or e-cigarette even one time? (Yes/No) Do you currently use e-cigarettes or electronic nicotine vapes every day, some days, or not at all?	0 = Reported either never using an e-cigarette/nicotine vape, or ever used an e-cigarette/nicotine vape but currently not at all. 1 = Currently uses e-cigarettes/nicotine vapes some days or every day.
**Combustible cigarette smoking**	Have you ever smoked a combustible tobacco cigarette, even just one puff? (Yes/No) Do you currently smoke cigarettes every day, some days, or not at all?	0 = Reported either never smoked a combustible cigarette, or ever smoked a combustible cigarette but currently not at all. 1 = Currently smokes combustible cigarettes some days or every day.

**Table 2.** Descriptive Statistics for Study Variables

	Sexual and Gender Minority (n = 1,288)		Heterosexual (n = 1,368)		

Measure	N(M)	%(SD)	N(M)	%(SD)	Χ^2^ (t)
	
**Race**					
White	829	64	757	55	25.87**
BIPOC^1^	459	36	629	45	
**Education**					
No 4-year degree	679	53	648	47	9.26**
4-year degree or higher	609	47	738	53	
**Age**					
18–24	543	42	384	28	60.93**
25–35	745	58	1002	72	
**Combustible current**					
Yes	333	26	426	31	7.59**
No	955	74	960	69	
**Vape current**					
Yes	455	35	499	36	0.10
No	833	65	887	64	
**Financial strain** ^2^	5.35	3.69	4.62	3.67	5.17**
**Food security** ^3^	4.24	3.70	3.62	3.61	4.43**
**Interpersonal discrimination** ^4^	11.98	4.84	10.47	4.69	8.24**
**Perceived stress** ^5^	8.61	3.56	7.26	3.60	9.71**

1. Black, Indigenous, People of Color

2. Range: 0–12

3. Range: 0–12

4. Range: 0–36

5. Range: 0–16

* p<0.05

** p<0.01

**Table 3.** Correlation among measured variables, Heterosexual Correlation among measured variables, SGM

	1	2	3	4	5	6	7	8	9	10	11	12	13
**1.Attitudes** ^1^	1												
**2.APP** ^2^	0.60**	1											
**3.DP** ^3^	0.19*	0.44**	1										
**4.FS** ^4^	0.01	0.06*	0.03	1									
**5.FI** ^5^	0.02	0.04	0.01	0.79**	1								
**6.ID** ^6^	−0.04	−0.01	−0.02	0.37**	0.38**	1							
**7.PS** ^7^	0.01	0.02	0.00	0.52**	0.46**	0.27**	1						
**8.Race** ^8^	0.01	−0.10**	−0.11**	−0.01	0.04	0.03	0.02	1					
**9.EDU** ^9^	−0.03	−0.08*	−0.10**	−0.17**	−0.21**	−0.05	−0.16**	0.05	1				
**10.Age** ^10^	−0.01	0.02	0.01	0.03	−0.05*	−0.09**	−0.05*	−0.09**	0.22**	1			
**11.Latinx** ^11^	−0.01	0.02	0.08*	-0.05	−0.07*	−0.02	−0.02	−0.42**	0.09**	0.06*	1		
**12.Combust** ^12^	−0.04	0.02	0.03	0.12**	0.13*	0.12**	0.07**	−0.08**	−0.09**	0.09**	−0.01	1	
**13.Vape** ^13^	0.03	0.02	0.02	0.11**	0.09**	0.12**	0.08**	−0.10**	−0.11**	−0.06*	0.05	0.32**	1
	1	2	3	4	5	6	7	8	9	10	11	12	13
**1.Attitudes** ^1^	1												
**2.APP** ^2^	0.58**	1											
**3.DP** ^3^	0.23**	0.41**	1										
**4.FS** ^4^	0.03	0.08*	0.05	1									
**5.FI** ^5^	0.03	0.05	0.06*	0.75**	1								
**6.ED** ^6^	−0.04	−0.01	0.04	0.36**	0.36**	1							
**7.PS** ^7^	−0.01	0.01	−0.03	0.49**	0.41**	0.37**	1						
**8.Race** ^8^	0.06*	−0.09**	−0.12**	−0.05	−0.01	0.01	−0.02	1					
**9.EDU** ^9^	−0.03	−0.11**	−0.10**	−0.08*	−0.18**	−0.06*	−0.12**	0.00	1				
**10.Age** ^10^	−0.05	−0.04	0.00	0.10**	0.01	−0.03	−0.04	−0.05	0.23**	1			
**11.Latinx** ^11^	−0.06*	0.03	0.15**	0.04	0.03	0.06*	0.02	−0.51**	0.03	0.09**	1		
**12.Combust** ^12^	−0.01	0.03	0.04	0.11**	0.15**	0.16**	0.05	0.03	−0.01	0.10**	0.02	1	
**13.Vape** ^13^	0.01	0.03	−0.01	0.10*	0.13**	0.11**	0.07*	−0.01	−0.04	−0.06*	0.03	0.32**	1

* p<0.05

** p<0.01

1. Attitudes towards sexually minoritized people

2. Absence of LGBTQ+ protective policy

3. LGBTQ+ Discriminatory policy that has been introduced or enacted

4. Financial strain

5. Food insecurity

6. Interpersonal discrimination

7. Perceived stress

8. Race coded 0 = BIPOC, 1 = white

9. Education coded 0 = no 4-year degree, 1= 4-year degree or higher

10. Age coded 0 = 18–24, 1 = 25–35

11. Latinx coded 0 = non-Latinx, 1 = Latinx

12. Current use of combustible cigarettes

13. Current use of nicotine vapes

* p<0.05

** p<0.01

1. Attitudes towards x

2 Absence of protective policy

3. Discriminatory policy that has been enacted

4. Financial strain

5. Food insecurity

6. Everyday discrimination

7. Perceived stress

8. Race coded 0 = BIPOC, 1 = white

9. Education coded 0 = no 4-year degree, 1= 4-year degree or higher

10. Age coded 0 = 18–24, 1 = 25–35

11. Latinx coded 0 = non-Latinx, 1 = Latinx

12. Current use of combustible cigarettes

13. Current use of nicotine vapes

**Table 4.** Indirect effects of structural stigma, economic depletion, and interpersonal discrimination on current use ofcombustible cigarettes or nicotine vapes.

Indirect pathway						

	SGM			Heterosexual		

	β	SE	Z	β	SE	Z
**Tobacco use**						
Effect of structural stigma via economic depletion	0.012	0.006	2.163*	0.005	0.003	1.590
Effect of structural stigma via interpersonal discrimination	−0.002	0.004	−0.463	0.000	0.005	0.002
Effect of economic depletion via perceived stress	−0.002	0.002	−1.339	−0.001	0.001	−1.053
Effect of interpersonal discrimination via perceived stress	0.000	0.000	0.443	0.000	0.000	−0.002
Effect of interpersonal discrimination via economic depletion	0.058	0.019	3.008**	0.041	0.019	2.123*

* p < 0.05

** p < 0.01

## Data Availability

Data and relevant code is available upon reasonable request from the PI (JGP).

## Abbreviations

BIPOC: Black, Indigenous, and People of Color
CFI: Comparative Fit index
MLE: Maximum Likelihood Estimation
RMSEA: Root Mean Square Error of Approximation
SEM: Structural Equation Modeling
SGMYA: Sexual and gender minority young adult
SGM: Sexual and gender minority
SRMR: Standardized Root Mean Squared Residual
YA: young adults
